# Gaussian Pseudorandom Number Generator Based on Cyclic Rotations of Linear Feedback Shift Registers

**DOI:** 10.3390/s20072103

**Published:** 2020-04-08

**Authors:** Guillermo Cotrina, Alberto Peinado, Andrés Ortiz

**Affiliations:** Departamento de Ingeniería de Comunicaciones, Universidad de Málaga, 29071 Málaga, Spain; gcotrinacuenca@uma.es (G.C.); aortiz@ic.uma.es (A.O.)

**Keywords:** LFSR, gaussian distribution, rotations, central limit theorem

## Abstract

This paper presents a new proposal to generate optimal pseudorandom numbers with Gaussian distribution. The generator is especially designed for low-cost hardware implementation, although the software version is also considered. For this reason, Linear Feedback Shift Registers in conjunction with cyclic rotations are employed. The proposal presents a low implementation cost and overcomes the limitations of the previous Gaussian generators based on linear feedback shift registers by means of a less complex algorithm to find optimal configurations. As a consequence, it turns into a really usable generator. Moreover, a further improvement, based on the simulated annealing algorithm, is applied in order for the random values to be better adjusted to the normal distribution.

## 1. Introduction

Linear feedback shift registers (LFSR) have always been a basic resource for the pseudorandom number generation (PRNG) due to their low cost implementation, the good statistical properties of the values produced and the simplicity of their mathematical model that allows a priori analysis of the behavior of the system [1]. The uniform distribution of the generated numbers allows LFSR to be widely used in communication and cryptographic applications, as part of the core of CDMA systems [2] and stream ciphers [3] belonging to the security standards and protocols of wireless and mobile telecommunication systems such as Bluetooth [4], IEEE 802.11 WLAN [5], GSM [6] and LTE [7]. LFSR are also employed to design true random number generators (TRNG) [8] in radio frequency identification (RFID) systems [9].

On the other hand, quantum key distribution schemes (QKD) are evolving from the initial discrete variable proposals (DV-QKD) [10] based on the transmission of polarized photons using non-orthogonal states towards continuous variable systems (CV-QKD) [11] based in the transmission of coherent states which allow the use of standard communications components and, therefore, lower implementation cost. CV-QKD schemes employ Gaussian modulation to send random amplitude and phase values that must be generated following a Gaussian distribution [12,13,14].

Although initially motivated by the potential cryptographic application, we explore in this paper the utilization of LFSR as a general purpose PRNG with Gaussian distribution instead of their native uniform distribution. Some authors have previously proposed Gaussian PRNG using LFSR. In 2010, Kang [15] presented a method employing an LFSR of length N=4M bits to generate pseudorandom numbers with (M+4) bits. The generation algorithm was based on an accumulator operated over decimated *M*-bits numbers, producing a final period of $\left(2^{N}-1\right)/\left(8N\right)$ which yields on an oversize LFSR. More recently, in 2015, Condo et al [16] have proposed a PRNG using permutations over the successive states of an LFSR. This generator, designed using a unique LFSR of length 17, reduces the cost of implementation. However, as the own authors claim, not all permutations can be applied. Furthermore, a high computational cost is required for the searching of valid permutations. Once these permutations have been applied in the PRNG, the numbers generated follow a Gaussian distribution according to the results of the normality tests. In other words, only when the tests results are greater than a given threshold, the permutation is considered a valid one.

All of these proposals are focused on the application of the central limit theorem (CLT) [17] that states that the distribution of samples mean approximates a normal distribution, as the sample size becomes larger, assuming that all samples are identical in size, and regardless of the population distribution shape. In this case, the samples produced by LFSR follow a uniform distribution. The use of several of these sequences leads us to obtain an approximation of a Gaussian distribution by means of the sum of all of them. Some authors [18] propose the utilization of several LFSR to generate different and independent uniform distributed sequences to be summed later. Other proposals Kang [15] and Condo [16] are based on a unique LFSR that produces all the sequences in order to decrease the global complexity of the PRNG.

Although the application of CLT is not the only method to generate Gaussian random numbers [19], it will always be a reference to take in mind. In [18], a comparison is performed among the hardware implementation of three of the best-known methods: CLT, Box–Muller algorithm [20,21] and polarization decision algorithm [22]. The comparison reveals that the number of gates and other hardware resources are very similar, while the CLT, implemented in a field-programmable gate array (FPGA) using directly the numbers produced by several LFSR, showed worse results in the normality tests.

However, the proposals based on a unique LFSR require a lower implementation cost. For this reason, we present in this article a much simpler implementation of the CLT method, mainly oriented to a hardware implementation, following the same strategy than Kang [15] and Condo et al [16], that is, using only one LFSR. The proposal requires the same resources than Condo et al’s PRNG but overcomes the oversize of Kang’s PRNG [15] and the inconvenient of Condo et al’s PRNG [16] related to the searching algorithm for valid configurations and reduces its computational cost. It is achieved by means of rotations, instead of generic permutations, reducing the complexity of precomputation performed to obtain the valid configurations (rotations). This fact turns the proposal into a really usable PRNG.

Next Section 1 and Section 2, describe the fundamentals of the LFSR and the previous proposals on CLT implementations based on LFSR. Section 3 presents the proposed PRNG based on LFSR rotations, while Section 4 and Section 5 contain the statistical tests applied to check the distribution of the generated numbers and the results of their application, respectively. In Section 6, a further improvement of the proposed scheme is presented by means of two coefficients, computed by the simulated annealing algorithm that helps the generated values to be better adjusted to the normal distribution. Conclusions are presented in Section 7.

## 2. Lfsr Fundamentals

**Figure 1:**
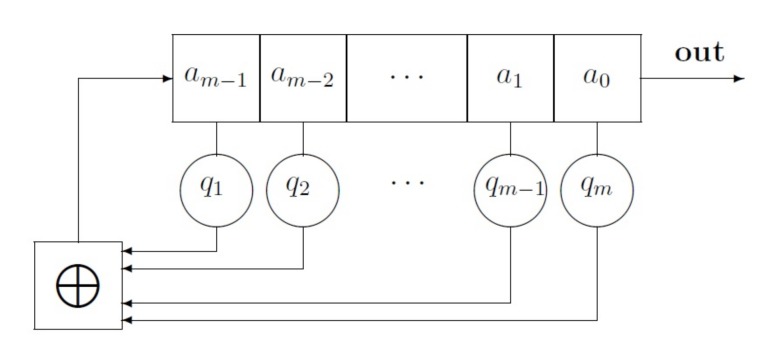
A Linear Feedback Shift Register of Length *m*.

In this section the basic properties of the LFSR (see Figure 1), and its generated sequences are described.

**Definition** **1.**  *(cf. [3]) A linear feedback shift register (LFSR) of length m consists of m stages numbered 0,1,2,⋯m-1, each capable of storing one bit and having one input and one output; and a clock which controls the movement of data. During each unit of time the following operations are performed:*

- *The content of stage 0 is output and forms part of the output sequence (* ***out*** *).*
- *The content of stage i is moved to stage i-1 for each i where 1≤i≤m-1.*
- *The new content content of state m-1 is the feedback bit $a_{j}$ which is calculated by adding together modulo 2 the previous contents of a fixed subset of stages 0,1,⋯,m-1.*

From this definition follows that the value $q_{i}$ is either 0 or 1 (Figure 1) and the feedback bit $a_{j}$ is the modulo 2 sum of the contents of those stages *i*, 1≤i≤m-1, for which $q_{m-i}=1$. As a consequence, the output sequence of the LFSR is $A=\left(a_{0},a_{1},a_{2},\cdots \right)$ and is uniquely determined by the following recursion:

(1) $$a_{j}=q_{1}\cdot a_{j-1}+q_{2}\cdot a_{j-2}+\cdots +q_{m}\cdot a_{j-m}$$

The behavior of the LFSR and the sequences generated can be performed by means of a polynomial whose coefficients are the values $q_{i}$ that represents the stages used to compute the feedback bit $a_{j}$. For this reason, the LFSR is denoted 〈m,p(x)〉, where $p\left(x\right)=1+q_{1}x+q_{2}x^{2}+\cdots +q_{m}x^{m}$ is the *connection polynomial*.

The LFSR is said to be *nonsingular* if the degree of p(x) is *m* (that is, $q_{m}=1$). If the initial content of stage *i* is $s_{i}\in \left\{0,1\right\}$ for each *i*, 0≤i≤m-1, then $\left[a_{m-1},\cdots ,a_{1},a_{0}\right]$ is called the *initial state* or *seed* of the LFSR.

On the other hand, the state of the LFSR at the time *t* is denoted as $s^{\left(t\right)}=\left[a_{m-1+t},\cdots ,a_{t+1},a_{t}\right]$, which corresponds to the application of the recursion in the Equation (1) *t* consecutive times starting with the seed $s^{\left(0\right)}=\left[a_{m-1},\ldots ,a_{1},a_{0}\right]$

**Example** **1.**  *Consider the LFSR $\langle 4,1+x+x^{4}\rangle$. If the initial state of the LFSR is $s^{\left(0\right)}=\left[0,0,0,0\right]$, the output sequence is the zero sequence $A=\left(0,0,\cdots \right)$. For the initial state $s^{\left(0\right)}=\left[0,1,1,0\right]$, the sequence has a length of 15. The Table 1 shows the successive states $s^{\left(t\right)}$. Note that the right-most bit of each state constitutes the output sequence $A=\left(0,1,1,0,0,1,0,0,0,1,1,1,1,0,1,0,\cdots \right)$.*

**Definition** **2.**  *(cf. [3]) An output sequence* ***A*** *$=\left(a_{0},a_{1},\cdots \right)$ generated by an LFSR 〈m,p(x)〉, is said to be periodic if there exits $j_{0}\in N$ such that $a_{i}=a_{i+j_{0}}$∀i∈N. Such $j_{0}$ is called period of the sequence.*

From this definition, the sequence of the example is a periodic sequence with period L=15.

One of the advantages of LFSR is the mathematical model that allows one to predict the length of the sequences generated. The following definition and theorem states how and when the maximal length is reached by the sequences.

**Definition** **3.**  *(cf. [3]) If $p\left(x\right)\in Z_{2}\left[x\right]$ is a connection polynomial of degree m, then 〈m,p(x)〉 is called a maximum length LFSR if the output sequence, with non-zero initial state, has period $2^{m}-1$. This sequence is called m-sequence.*

**Theorem** **1.**  *(cf. [1]) An output sequence* ***A*** *generated by an LFSR 〈m,p(x)〉 is an m-sequence if and only if the connection polynomial p(x) is a primitive polynomial. The sequence length is independent of the initial state.*

Consequently, a primitive polynomial of degree *m* will generate a sequence of length $2^{m}-1$ and the LFSR will run through $2^{m-1}$ different nonzero states, that is, all possible nonzero states. Hence, if we consider each state as an *m*-bit pseudorandom number, we can say that LFSR produce numbers with uniform distribution.

Besides its maximal length, the *m*-sequences have many desirable statistical properties that can be summarized in the three Golomb’s postulates [1]. Given a periodic binary sequence $A={\left(a_{i}\right)}_{i\in N}$ with period length $L=2^{m}-1$, it is said to be pseudoradom if the following postulates hold.

1. Distribution test. In every period, the number of ones is nearly equal to the number of zeros, more precisely the difference between the two numbers is at most 1:
  (2) $$\left|\sum_{i=1}^{L}{\left(-1\right)}^{a_{i}}\right|\leq 1$$
2. Serial test. A sequence of consecutive ones is called a block and a sequence of consecutive zeros is called a gap. A run is either a block or a gap. In every period, one half of the runs has length 1, one quarter of the runs has length 2, and soon, as long as the number of runs indicated by these fractions is greater than 1. Moreover, for each of these lengths the number of blocks is equal to the number of gaps.
3. Autocorrelation test. The auto-correlation function
  (3) $$C\left(\tau \right)=\sum_{i=0}^{L-1}{\left(-1\right)}^{a_{i}}{\left(-1\right)}^{a_{1+\tau }}$$
  is two-valued.

## 3. Gaussian Generators Based on Lfsr

Several authors [15,16,18] have proposed the utilization of LFSR to generate random numbers with Gaussian distribution performing direct implementations of the CLT, that is, producing several sequences of uniform distributed random numbers that are then summed to approximate to the normal distribution.

In order to obtain low cost implementations, Kang [15] in 2010 and Condo [16] in 2015 have proposed PRNG with only one LFSR. Kang’s proposal uses one LFSR to generate 4 different sequences of numbers that are summed to produce the final Gaussian random value. To do that, the state of an LFSR of N=4M bits is splitted into 4M-bit numbers that are summed. The result of the addition is stored in an accumulator. *N* clock cycles later the LFSR state is splitted again to produce a new input into the accumulator. This operation is repeated 8 times to finally obtain a (N+4)-bit pseudorandom number at the accumulator output. This numbers follow a Gaussian distribution. However, the PRNG is not efficient due to the oversizing required for the LFSR.

**Figure 2:**
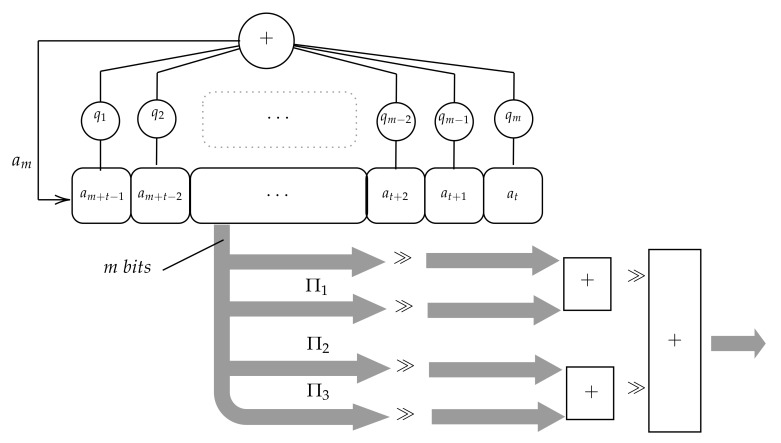
Scheme of the Gaussian generator proposed by Condo and Gross.

In 2015, Condo et al. [16] proposed also a Gaussian PRNG using only one LFSR. In this case, instead of splitting the state, the system generates several uniform distributed sequences applying several permutations to every LFSR state. More precisely, two PRNG versions are proposed in [16]. The first one, depicted in Figure 2, produces four sequences of numbers or, in other words, four numbers at every instant *t*: the LFSR state $s^{\left(t\right)}$ and three additional numbers obtained applying three different permutations $\pi_{i}$, $\pi_{j}$, $\pi_{k}$ to $s^{\left(t\right)}$. The second version produces only 3 sequences of numbers because it only applies two permutations $\pi_{i}$, $\pi_{j}$ to every state $s^{\left(t\right)}$.

The sequences generated by the two versions have been analyzed in [16] using only the LFSR $\left\{17,x^{17}+x^{14}+1\right\}$. However, the authors in [16] have provided an estimation of the implementation cost for a generic PRNG with an LFSR of *N* stages. This generic design requires one N-bit register, 3N-bit adders (or 2N-bit adders for the second version) and J XOR gates, J being the number of stages to implement the LFSR feedback. Note also that permutations can be implemented by scrambling the order of the wires connecting the LFSR to the adder. Hence, they do not require additional hardware resources, such as gates or registers, thus helping to not increase the total implementation cost. As a result, this PRNG has lower cost than Kang’s PRNG [15]. In order to generate *N*-bit random numbers, the Kang’s PRNG requires one LFSR with 4(N-4) stages, 3(N-4)-bit adders and J XOR gates.

Despite the low implementation complexity, this generator has some drawbacks:

- According to the authors only 1/15 of the permutations sets $\left\{\pi_{i},\pi_{j},\pi_{k}\right\}$ produce a Gaussian distribution output. Moreover the set of such permutations is not characterized, which implies the necessity of having to perform an exhaustive search to choose them. This proposed method in [16] requeries the generation of the complete sequence for each possible combination.
- According to the authors, the set of valid permutations depends on the chosen seed, that greatly complicates its practical application.

## 4. Gaussian Generator Based on Lfsr Rotations

This section describes the proposed generator that follows the same approach as in [16]; i.e., the implementation of the CLT applied over samples with uniform distribution generated by means of only one LFSR. The main difference is that all sequences of numbers are generated from the unique LFSR by applying cyclic rotations (a particular case of permutations) instead of the generic permutations proposed in [16]. The rotation is just a cyclic shift of the content of a given state of the LFSR. Considering the state as a binary vector, the rotation implies the shift to the right of every component. The right-most component is then moved to the left-most one. A k-rotation implies *k* single rotations. The rotations are always applies to the right. As a consequence, since the rotations, as well as permutations, can be implemented by scrambling the wires connecting the LFSR to the adder, the implementation cost is the same, one N-bit register, 3 or 2N-bit adders and J XOR gates. However, the percentage of rotations that produce Gaussian random numbers is much greater than that of generic permutations. This fact allows one to randomly select the rotations with a high probability that they can be applied in the Gaussian generation. In this way, we solve the main drawback of the Condo et al PRNG.

The proposed generator is also designed in two different versions (the first using three rotations; the second only two) in order to facilitate the comparison to the PRNG in [16]. In both versions, the LFSR is defined by a primitive polynomial p(x), hence producing an m- sequence.

**Figure 3:**
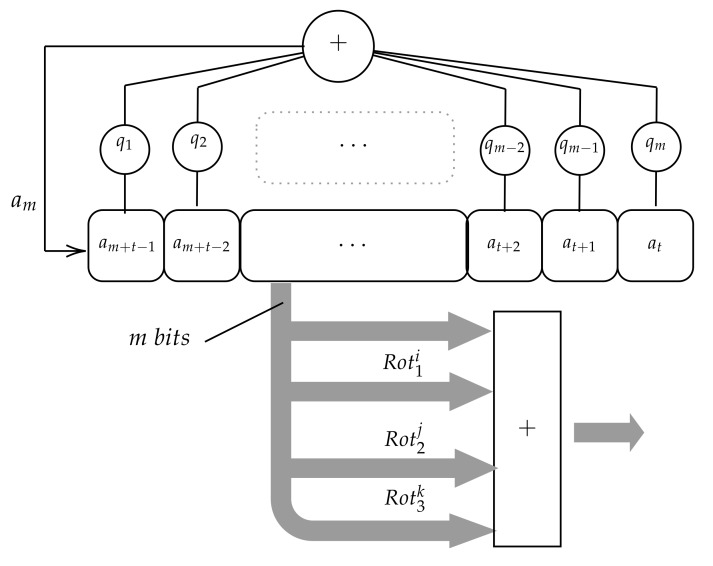
Gaussian generator based on LFSR rotations.

In Figure 3, the first version is shown, in which the LFSR runs over $2^{m}-1$ states. At every clock pulse t the state $s^{\left(t\right)}$ is then considered as an *m*-bit number and added to other three m-bit numbers produced by applying three rotations to the state $s^{\left(t\right)}$. The rotations are defined as follows.

Let’s consider an LFSR $\langle m,p(x)\rangle$ where p(x) is a primitive polynomial of degree *m* in order to produce an m-sequence, according to Theorem 1. For every LFSR state $s^{\left(t\right)}$ a rotation function $Rot^{\left(k\right)}$ is defined as the cyclic *k* shifts to the right of the state content. Hence, as $s^{\left(t\right)}=\left[a_{m-1+t},\cdots ,a_{1+t},a_{t}\right]$ we have

(4) $$\begin{array}{c} Rot^{\left(1\right)}\left(s^{\left(t\right)}\right)=Rot^{\left(1\right)}\left(\left[a_{m-1+t},\cdots ,a_{1+t},a_{t}\right]\right)=\left[a_{t},a_{m-1+t},\cdots ,a_{1+t}\right]\\ Rot^{\left(2\right)}\left(s^{\left(t\right)}\right)=Rot^{\left(2\right)}\left(\left[a_{m-1+t},\cdots ,a_{1+t},a_{t}\right]\right)=\left[a_{1+t},a_{t},a_{m-1+t},\cdots ,a_{2+t}\right]\\ \cdots \end{array}$$

Note that $Rot^{\left(m\right)}\left(s^{\left(t\right)}\right)=s^{\left(t\right)}$. We denote $Rot_{1}^{\left(j\right)},Rot_{2}^{\left(k\right)},Rot_{3}^{\left(l\right)}$ the three rotations applied to the state $s^{\left(t\right)}$ in the first version of the PRNG, with j≤k≤l without loss of generality. Similarly, we denote $Rot_{1}^{\left(j\right)}$, $Rot_{2}^{\left(k\right)}$ the two rotations applied to the state $s^{\left(t\right)}$ in the second version of the PRNG, with j≤k without loss of generality.

Finally, the random number $RN^{\left(t\right)}$ produced at time *t* by this generator is computed as follows:

(5) $$\tau^{\left(t\right)}=D\left(s^{\left(t\right)}\right)+D\left(Rot_{1}^{j}\left(s^{\left(t\right)}\right)\right)+D\left(Rot_{2}^{k}\left(s^{\left(t\right)}\right)\right)+D\left(Rot_{3}^{l}\left(s^{\left(t\right)}\right)\right)$$

where *D* is the function that maps an m-bit vector into a decimal value, that is,

(6) $$D\left(s^{t}\right)=\sum_{i=0}^{m-1}2^{i}\cdot a_{t+i}$$

It is also important to note that the sequence generated by the LFSR is always of length $2^{m}-1$ and independent from the seed, provided that p(x) is primitive and the seed is a nonzero state. This fact allows the utilization of any primitive polynomial and any nonzero seed and, hence, the turns the PRNG into a real usable one.

We should take into consideration the fact that these equations only appear in order to keep the mathematical formalism but it has not to be implemented in hardware since the electronic components works directly with the binary representation of the numbers.

It is also important to highlight that the sequence generated by the LFSR is always of length $2^{m}-1$ and independent from the seed, provided that p(x) is primitive and the seed is a nonzero state. This fact allows the utilization of any primitive polynomial and any nonzero seed and, hence, the turns the PRNG into a real usable one.

## 5. Statistical Analysis

In this section, the distribution of the numbers generated by the proposed PRNG is analysed. Several normality tests have been applied to identify the configurations (sets of three or two rotations) that generates numbers with Gaussian distribution.

### 5.1. Distribution Fit Test

A distribution fit test performs a goodness of fit hypothesis test with null hypothesis $H_{0}$ that data was drawn from a population with a specific distribution of values, in this case the Normal distribution, and alternative hypothesis that it was not. A statistical hypothesis test returns a value called *p* or the *p*-value. This value is used to reject or fail to reject the null hypothesis. This is done by comparing the *p*-value to a threshold value chosen beforehand called the significance level α. When the *p*-value is less than α, the default hypothesis can be rejected. In the same way, the confidence level of the test is 1-α. If we set the significance level to 5% and the *p*-value is greater than 95%, we would conclude that the null hypothesis affirming that the data is distributed according to the Normal Distribution would not be rejected at the 5 percent significance level. In the present context, the higher the *p*-value, the better the data fits the normal distribution.

There exist different methods to distinguish whether or not the range of values in a distribution follows a Normal distribution. In Table 2, the normality tests we have considered during the analysis are shown. In the next subsection we described the most relevant ones. The table shows the results obtained in the application of the test to the numbers generated when 3 particular rotations are implemented.

In order to apply the tests, we define the statistical variable to be analyzed as

(7) $$X_{\left(i,j,k\right)}=\tau \left(s^{n}\right),\forall n\in \left\{0,1,\cdots ,2^{m}-1\right\}$$

in the case of 3 rotations model and as

(8) $$X_{\left(i,j\right)}=\tau \left(s^{n}\right),\forall n\in \left\{0,1,\cdots ,2^{m}-1\right\}$$

in the case of a 2 rotations model.

According to the CLT [23], if we consider $\left\{X_{1},\ldots ,X_{n}\right\}$ a random sample of size *n* that is, a sequence of independent and identically distributed random variables drawn from a distribution of expected value given by μ and finite variance given by $\sigma^{2}$. Suppose we are interested in the sample average $S_{n}=\frac{X_{1}+\cdots +X_{n}}{n}$ of these random variables. By the law of large numbers, the sample averages converge in probability and almost surely to the expected value μ as n⟶∞. The classical central limit theorem describes the size and the distributional form of the stochastic fluctuations around the deterministic number μ during this convergence. More precisely, it states that as n gets larger, the distribution of the difference between the sample average $S_{n}$ and its limit μ, when multiplied by the factor $\sqrt{n}$ (that is $\sqrt{n}\left(S_{n}-\mu \right)$ ), approximates the normal distribution with mean 0 and variance $\sigma^{2}$. For large n, the distribution of $s_{n}$ is close to the normal distribution with mean μ and variance $\sigma^{2}/n$. The usefulness of the theorem is that the distribution of $\sqrt{n}\left(S_{n}-\mu \right)$ approaches normality regardless of the shape of the distribution of the individual Xi.

**Figure 4:**
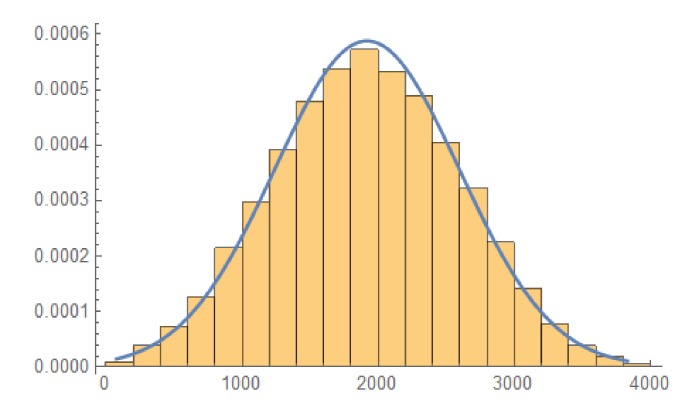
Histograms where a three rotations model has been applied over an LFSR generated controlled by a primitive polynomial of degree m=10.

In Figure 4 we can visualize the histogram of $X_{\left(2,5,8\right)}$ for 3 rotations versions where the polynomial p(x) is primitive of degree m=10 and $Rot_{1}^{2},Rot_{2}^{5},Rot_{3}^{8}$ have been applied.

#### 5.1.1. Anderson-Darling Test

The Anderson-Darling [24] test is used to test if a sample of data came from a population with a specific distribution. It is a modification of the Kolmogorov-Smirnov (K-S) [24] test that endives more weight to the tails than does this test. The K-S test is distribution free in the sense that the critical values do not depend on the specific distribution being tested. The Anderson-Darling test makes use of thespecific distribution in calculating critical values. This has the advantage of allowing a more sensitive test and the disadvantage that critical values must be calculated for each distribution. Currently, tables of critical values are available for the normal, uniform, lognormal, exponential, Weibull, generalized Pareto, and logistic distributions. The Anderson-Darling test is an alternative to the chi-square [24] and Kolmogorov-Smirnov goodness of fit tests.

The definition is as follows:

$H_{0}:$ the data follows a specific distribution, $H_{a}:$ The data do not follow the specified distribution. The test is defined as:

(9) $$A^{2}=-N-S$$

where

(10) $$S=\sum_{i=1}^{N}\frac{\left(2i-1\right)}{N}\left[ln(F\left(Y_{i}\right)+ln(1-F\left(Y_{N+1-i}\right)\right]$$

where *F* is the cumulative distribution function of the specified distribution, $Y_{i\in N}$ is the set of values to be tested and *N* is the set of the stardard normal values.

The critical values for the Anderson-Darling test are dependent on the specific distribution that has been tested.

#### 5.1.2. Shapiro-Wilk Test

The Shapiro-Wilk test [25], calculates a W statistic that tests whether a random sample, $x_{1},x_{2},\cdots ,x_{n}$ comes from a normal distribution. Small values of W are evidence of departure from normality and percentage points for the W statistic, obtained via Monte Carlo simulations, were reproduced by Pearson and Hartley [25]. This test has done very well in comparison studies with other goodness of fit tests.

The W statistic is calculated as follows:

(11) $$W=\frac{{\left(\sum_{i=1}^{n}\alpha_{i}x_{\left(i\right)}\right)}^{2}}{\sum_{i=1}^{n}x_{i}-{\bar{x}}^{2}}$$

where the x(i) are the ordered sample values (x(1) is the smallest) and the $\alpha_{i}$ are constants generated from the means, variances and covariances of the order statistics of a sample of size *n* from a normal distribution.

#### 5.1.3. Chi Square Test

The chi-square test [24] is used to test if a sample of data came from a population with a specific distribution. In this case we shall focus on this test to check if the distribution of numbers fits the normal distribution.

An attractive feature of the chi-square goodness-of-fit test is that it can be applied to any univariate distribution for which you can calculate the cumulative distribution function. The chi-square goodness-of-fit test is applied to binned data (i.e., data put into classes). This is actually not a restriction since for non-binned data you can simply calculate a histogram or frequency table before generating the chi-square test.

The chi-square test is an alternative to the Anderson-Darling and Kolmogorov-Smirnov goodness-of-fit tests, that is much better in terms of the tale analysis, given that this is esential for the Gaussian noise emulator.

The chi-square test is defined for the hypothesis:

$H_{0}:$ The data follow a normal distribution.

$H_{1}:$ The data do not follow the specified distribution.

Test Statistic: For the chi-square goodness-of-fit computation, the data are divided into k bins and the test statistic is defined as:

(12) $$\chi^{2}=\sum_{i=1}^{k}\frac{{\left(O_{i}-E_{i}\right)}^{2}}{E_{i}}$$

where $O_{i}$ is the frequency of the i-th value and $E_{i}$ its corresponding frequency.

In order to deploy this test we firstly assume that $x_{1},x_{2},...,x_{n}$ are the observed values of a variable *x*. Then we continue the following steps.

1. Categorize the observations (n) into *k* categories.
2. Calculate the frequencies $f_{i}$, i∈{1,2,⋯,k}, where each $f_{i}$ is the observed frequency of the category *i*.
3. Let $p_{i}$ be the probability, that under null hypothesis, the random variable *x* belongs to the category *i*. Then we calculate the expected frequencies $E_{i}=np_{i}$ of the observations in category *i*.
4. Under the null hypothesis, Note that the random variables $f_{1},f_{2},\cdots ,f_{k}$ follow multinomial distribution with parameters $n,p_{1},p_{2},...,p_{k}$.
5. Calculate the test statistic $\chi_{g}^{2}=\sum_{i=1}^{k}\frac{{\left(O_{i}-E_{i}\right)}^{2}}{E_{i}}$. The expected value of the test statistic, under the null hypothesis, k-1-e. ( That is $E\left[\chi_{g}^{2}\right]=k-1-e$.)
6. Large and small values of the test statistic (compared to the expected value) suggest that the null hypothesis $H_{0}$ does not hold.
7. If the *p*-value is small enough, the null hypothesis $H_{0}$ is rejected.

### 5.2. Results and Comparison with Existing Models

These described tests have been used to check whether a set of values $X_{\left(i,j,k\right)}$ defined as in the Equation (7), is distributed according to a Normal distribution. A minimum level of confidence has been set to 90% therefore the significance level is set to 10% and according to that level of confidence the sequence obtained has been screened. That is, given a set of values, the Normality tests have been applied to verify whether the data followed a normal distribution or not. The output of these mentioned tests is a *p*-value. If the *p*-value obtained is greater than 90%, the sequence obtained is considered ***valid*** and otherwise has been discarded.

The proposed generator has been tested for all possible combinations $\{Rot_{1}^{j}$, $Rot_{2}^{k}\}$ where 0≤j<k≤m in the case of two rotations and $\{Rot_{1}^{j}$, $Rot_{2}^{k}$, $Rot_{3}^{l}\}$ where 0≤j<k<l≤m in the case of three that generates a Gaussian distribution model. All combinations have been tested using the Mathematica environment, for LFSR whose conection polynomials have degrees 6≤m≤22. The tests have been performed using Chi sqaure, the Anderson-Darling and Shapiro methods. The results are summarize in Table 3 where for each degree *n*, 6≤n≤22 of the polynomials, the total number of existing rotations has been computed compared to the total number of valid rotations where the *p*-values has exceeded the established minimum of 90%.

It proves that the total number of combinations that can be formed considering groups of 3 cyclical rotations, and the percentages over the total number which generates a final sequence and it fits perfectly to a Gaussian distribution. Given that the rotation of *n* bits is equivalent to not apply any rotation, the total number can be worked out as:

(13) $$C_{m-1}^{3}=\frac{(m-1)!}{3!(m-4)!}.$$

**Figure 5:**
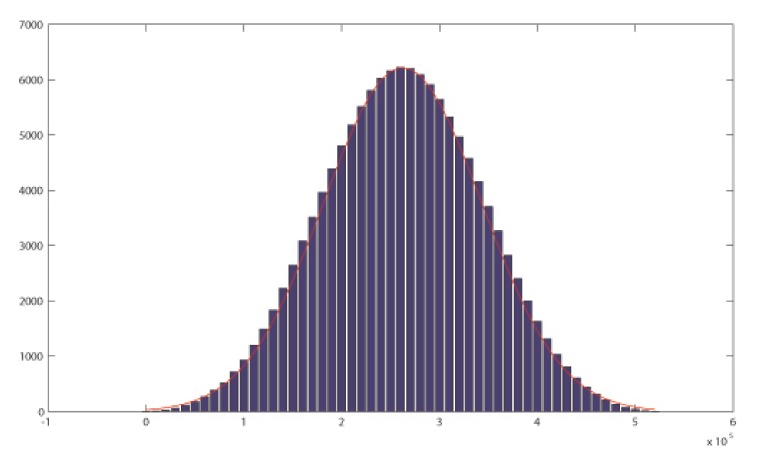
Distribution where n=17, three rotations applied.

The Figure 5 shows the histogram for a particular case where the 3 rotations model has been applied and the primitive polynomial has degree m=17.

As shown in the Table 3, the rotations allow a practical generator use, since the election of the set of rotations $Rot_{1}^{i}$, $Rot_{2}^{j}$, $Rot_{3}^{k}$ is much simpler than the election of permutations proposed by Condo in [16]. The election of the seed as well as the election of the primitive feedback polynomial are not relevant. Furthermore, a much higer percentage of rotations, 100 % in some cases, generates a Gaussian distribution. Valid permutations suggested by Condo in [16] represents only 6.6% of the total.

The cardinal of the set of valid combinations of two rotations $Rot1^{i},Rot2^{j}$, are also presented in Table 3, considering that the total number of combinations is:

(14) $$C_{m-1}^{2}=\frac{(m-1)!}{2!(m-3)!}$$

As in the case of three rotations, the Table 3 indicates a much higher percentage of 90% of all combinations of two rotations generating an output sequence with Gaussian distribution, for any *m*, length of the LFSR. The effect of rotations remains also independent of the seed and the primitive polynomial feedback chosen.

Finally, it is important to notice that the cost of implementation is the same as the generator Condo [16], since the same type of operations and the same number of adders are used.

The proposed PRNG [21] has also been compared with the Box-Muller algorithm that was designed as a pseudo-random number sampling method for generating pairs of independent, standard, normally distributed (zero expectation, unit variance) random numbers, given a source of uniformly distributed random numbers. If $U_{1}$ and $U_{2}$ are independent samples chosen from the uniform distribution on the unit interval (0,1), then the variables defined as:

(15) $$Z_{0}=Rcos\left(\Theta \right)=\sqrt{-2ln(U_{1})}\cdot cos\left(2\pi U_{2}\right)$$

(16) $$Z_{1}=Rsin\left(\Theta \right)=\sqrt{-2ln(U_{1})}\cdot sin\left(2\pi U_{2}\right)$$

are independent random variables with a standard normal distribution.

After having executed the Box-Muller algorithm we have found the following disadvantages.

- According to the results presented in Table 4, we can see that the values of the *p*-test are better in the 3 rotation model, than in the Box-Muller algorithm.
- The computational cost required to implement the algorithm is much higher.

## 6. Improvements in the Results Obtained

In previous sections, we have shown that the proposed PRNGs imrove the results obtained by Condo [16] and by Kang [15]. The proposed PRNG has been designed as a direct particularization of Condo’s system, using only rotations instead of a generic permutation, trying to obtain the easiest solution (with the minimum modification) to the problems detected in [16]. Nevertheless, the accuracy of the distribution in the proposed PRNG can be further improved, that is, the *p*-values can be increased. Since the results are more stable in the version of three rotations we have decided to build on this version. Though the number of valid rotations is more or less the same, a substantial improvement in terms of their accuracy has been obtained. For this, an LFSR based on a primitive polynomial has been considered.

We consider a LSFR controlled by a primitive polynomial of degree *m*, 〈m,p(x)〉. Let $s^{\left(t\right)}=\left[a_{m-1+t},a_{m-2+t},\cdots ,a_{t+1},a_{t}\right]$ be a state of the LFSR. Then we define the projections $\Pi_{1}$ and $\Pi_{2}$ as follows

(17) $$\Pi_{1}\left(s^{t}\right)=\left[0,a_{m-2+t},\cdots ,a_{t+1},0\right]$$

and

(18) $$\Pi_{2}\left(s^{t}\right)=\left[0,0,a_{m-3+t},\cdots ,a_{t+2},0,0\right]$$

Then, the random number is now generated as follows:

(19) $$\begin{array}{c} \tau \left(s^{t}\right)=\\ =D\left(Rot_{1}^{0}\left(s^{t}\right)\right)+D\left(Rot_{2}^{i}\left(s^{t}\right)\right)+D\left(Rot_{3}^{j}\left(s^{t}\right)\right)+D\left(Rot_{4}^{k}\left(s^{t}\right)\right)+\\ +D\left(\pi_{1}\left(Rot_{4}^{k}\left(s^{t}\right)\right)\right)+D\left(\pi_{2}\left(Rot_{4}^{k}\left(s^{t}\right)\right)\right) \end{array}$$

where the function *D* is defined in the Equation (5).

**Figure 6:**
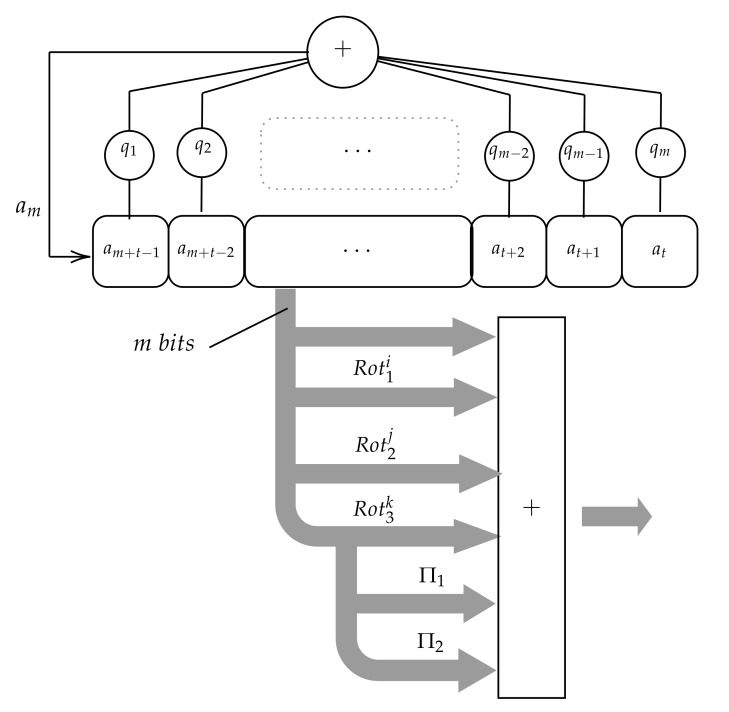
Improvements Gaussian generator based on LFSR rotations.

Figure 6 shows the implementation of this improvement. As one can see, the projections do not increase the implementation cost as they do not need gates or registers.

We have analyzed for all *n* that verifies 6≤n≤22. The number of valid rotations and variables is similar to those of the previous model, however the acceptance minimum level has been set to 95% which corresponds to a maximum estimation error below the treshold of 5%. The Table 5 shows that values that have been achieved.

In this particular case where the projections have been applied, we have tested that, although the acceptance level has been set to 95% we obtain more or less the same number of valid rotations. In other words, we have not only found a method that exceeds the models proposed to date, but also that the system has been refined to adapt the set of values to a Normal distribution. Although the system has given good experimental results, we have been able to verify that when we have increased the value of the degree of the polynomial in order to obtain a greater number of observations, then there was a decrease in the number of valid rotations.

Once these results have been obtained, we have tried to find a method that allows us to obtain a similar number of valid rotations similar to that obtained in the previous section and in the same way, if possible, increase the efficiency of the system. For this, we have used the simulated annealing method. The Simulated annealing method [26] is a method for solving unconstrained and bound-constrained optimization problems. The method models the physical process of heating a material and then slowly lowering the temperature to decrease defects, thus minimizing the system energy.

At each iteration of the simulated annealing algorithm, a new point is randomly generated. The distance of the new point from the current point, or the extent of the search, is based on a probability distribution with a scale proportional to the temperature. The algorithm accepts all new points that lower the objective, but also, with a certain probability, points that raise the objective. By accepting points that raise the objective, the algorithm avoids being trapped in local minima, and is able to explore globally for more possible solutions. An annealing schedule is selected to systematically decrease the temperature as the algorithm proceeds. As the temperature decreases, the algorithm reduces the extent of its search to converge to a minimum.

To illustrate how this method has been implemented, we will illustrate the procedure through an example. If we take the case where the degree of the polynomial n=12, we start with vectors of dimension 12 in which we are applying the method of rotations described in the previous section. Once this method has been applied, it has been determined which of the possible rotations has the highest coefficient and therefore a distribution of values closer to the normal distribution. In this case the values to be taken are those of $Rot_{1}^{2}$, $Rot_{2}^{4}$, $Rot_{3}^{8}$, $\Pi_{1}\left(Rot_{3}^{8}\right)$, $\Pi_{2}\left(Rot_{3}^{8}\right)$. If we use this combination of rotations, a *p* test value close to 97.5% is obtained. From this point on, this distribution of values will be taken as a fixed reference.

Once this distribution of values has been fixed, a function has been defined in which the values obtained by the projections, defined in Equations (17) and (18), are multiplied by two coeffcients α and β. Then the values have been obtained by working out the decimal sum defined by:

(20) $$\begin{array}{c} D\left(s^{\left(t\right)}\right)+D\left(Rot_{1}^{2}\left(s^{\left(t\right)}\right)\right)+D\left(Rot_{2}^{4}\left(s^{\left(t\right)}\right)\right)+D\left(Rot_{3}^{8}\left(s^{\left(t\right)}\right)\right)+\\ +\alpha \cdot D\left(\Pi_{1}\left(Rot_{3}^{8}\left(s^{\left(t\right)}\right)\right)\right)+\beta \cdot D\left(\Pi_{2}\left(Rot_{8}\left(s^{\left(t\right)}\right)\right)\right) \end{array}$$

where the function *D* is defined in the Equation (5).

In order to apply the method, a function has been defined that depends on the sequence and the parameters of α and β and whose output 1- will be the normal *p*-test value. This function is to which we will apply the simulated annealing method in order to minimize the value of this function. In the case where n=12 it has been obtained that α→1.48289,β→3.16175. There has been a significant increase in the number of valid rotations and in the same way it has been achieved that they are much closer to the Normal distribution.

For other values of *n* as is the case in which it takes the values of n={10,11} are obtained following the same method the values for α and β are: α→0.882965 and β→4.53362 for n=10 and α→1.30476 and β→3.64635 for the case where n=11, the remaining cases will be considered for future study.

**Figure 7:**
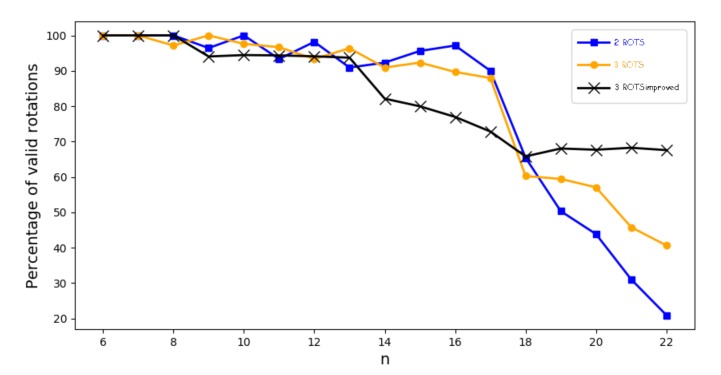
Evolutions of the percetages of valid rotations for the different models according to the degree of the primitive polynomial.

In Figure 7 we can observe the evolution of the percentage of valid rotations for each degree of the primitive polynomial and for each model (2 rotations, 3 rotations and 3 rotations with 2 projections). As seen on the trend line with squares, representing the tendency for the 2 rotations model, the percentage of valid rotations decreases as we increase the degree of the primitive polynomial. The situation improves in the case of the trend line with circles where the 3 rotations model is analyzed. For primitive polynomials with small degree the situation is acceptable, however as the degree of the polynomial increases, the percentage of valid rotations decreases. This decrease is not as noticeable, however, adequate values are not obtained. Finally, in the trend line of the crosses representing the case of the 3 rotations and the 2 projections, it is observed that the percentages of valid rotations are acceptable and constant, even for large values of the degree of the primitive polynomial.

## 7. Conclusions

This paper proposed a new pseudorandom number generator with Gaussian distribution using a method that reduces the cost of implementation, as it applies to [16]. The core difference is in the characterization of valid configurations. While in [16] the proposal is the use of permutations and to make a preliminary exhaustive search of them, depending on the seed and polynomial feedback, this paper proposes the use of a subset of these permutations, cycling rotations, concluding that more than 90% of the combinations of such rotations are suitable for its practical use. Furthermore, the generator is independent of the seed and the polynomial feedback once the length LFSR is fixed. Furthermore, additional projections have been applied to the initial design yielding numbers with better results in the normality tests without increasing the implementation cost. Finally, the simulated annealing algorithm has been applied to optimize the results obtained in the tests.

## Figures and Tables

**Table 1:** Values of the LFSR $\langle 4,1+x+x^{4}\rangle$ whose initial state is $\left[0,1,1,0\right]$.

*t*	$s^{\left(t\right)}$					*t*	$s^{\left(t\right)}$			
0	0	1	1	0		8	1	1	1	0
1	0	0	1	1		9	1	1	1	1
2	1	0	0	1		10	0	1	1	1
3	0	1	0	0		11	1	0	1	1
4	0	0	1	0		12	0	1	0	1
5	0	0	0	1		13	1	0	1	0
6	1	0	0	0		14	1	1	0	1
7	1	1	0	0		15	0	1	1	0

**Table 2:** *p*-tests values for a 3 Rotations model depicted in Figure 3, where n=17 and $Rot_{1}^{2},Rot_{2}^{6},Rot_{3}^{8}$ have been applied.

Normal Distribution Fit Test	Statistic	*p*-Value
Anderson-Darling	0.194777	0.901172
Cramér-von Mises	0.0269862	0.896351
Jarque-Bera ALM	4.00919	0.130367
Kolmogorov-Smirnov	0.0109818	0.995996
Kuiper	0.0200591	0.993069
Mardia Combined	4.00919	0.130367
Mardia Kurtosis	−2.01337	0.0440758
Mardia Skewness	0.0502429	0.822641
Pearson $\chi^{2}$	4.97263	1.0
Shapiro-Wilk	0.998453	0.504076
Watson $U^{2}$	0.0268772	0.863961

**Table 3:** Number of Valid Rotations at a confidence level of 90%.

LFSR	$\{{\mathrm{Rot}}_{1}^{j}$, ${\mathrm{Rot}}_{2}^{k}$, ${\mathrm{Rot}}_{3}^{l}\}$		$\{{\mathrm{Rot}}_{1}^{j}$, ${\mathrm{Rot}}_{2}^{k}\}$	
*n*	Total	Valid(%)	Total	Valid(%)
6	10	100	10	100
7	20	100	15	100
8	35	97.14	21	100
9	56	100	28	96.43
10	84	97.62	36	100
11	120	96.67	45	93.33
12	165	93.33	55	98.18
13	220	96.36	66	90.91
14	286	90.91	78	92.31
15	364	92.31	91	95.60
16	455	89.67	105	97.14
17	560	87.96	120	90
18	680	60.26	136	65.44
19	816	59.44	153	50.33
20	969	57.07	171	43.86
21	1140	45.79	190	31.05
22	1540	40.65	210	20.95

**Table 4:** Evolution of the *p*-values obtained, after applying the Chi Square Goodness of Fit Test method to a set of values, for Box-Muller and a 3 rotations LFSR model.

Polynomial Degree	Number of Values	*p*-Values for Box Muller	*p*-Values for an LFSR with 3 Rotations
n=6	$2^{6}=64$	p=0.92	p=0.99
n=7	$2^{7}=128$	p=0.91	p=0.98
n=8	$2^{8}=256$	p=0.91	p=1
n=9	$2^{9}=512$	p=0.90	p=1
n=10	$2^{10}=1024$	p=0.89	p=0.99
n=11	$2^{11}=2028$	p=0.86	p=0.99
n=12	$2^{12}=4096$	p=0.85	p=0.99
n=13	$2^{13}=8192$	p=0.85	p=0.98
n=14	$2^{14}$ = 16,384	p=0.83	p=0.97
n=15	$2^{15}$ = 32,768	p=0.83	p=0.95
n=16	$2^{16}$ = 65,536	p=0.82	p=0.93
n=17	$2^{17}$ = 131,072	p=0.81	p=0.92
n=18	$2^{18}$ = 262,144	p=0.81	p=0.92
n=19	$2^{19}$ = 524,288	p=0.78	p=0.92
n=20	$2^{20}$ = 1,048,576	p=0.77	p=0.91
n=21	$2^{21}$ = 2,197,152	p=0.76	p=0.91
n=22	$2^{22}$ = 4,194,394	p=0.76	p=0.90

**Table 5:** Number of Valid Rotations for an acceptance minimum of level 95% where the proyections $\Pi_{1}$ and $\Pi_{2}$ have been applied.

LFSR	$\left\{{\mathrm{Rot}}_{1}^{i},{\mathrm{Rot}}_{2}^{j},{\mathrm{Rot}}_{3}^{k},\Pi_{1}\left({\mathrm{Rot}}_{3}^{k}\right),\Pi_{2}\left({\mathrm{Rot}}_{3}^{k}\right)\right\}$	
n	**Total**	**Valid (%)**
6	60	100
7	105	100
8	168	100
9	252	94.04
10	360	94.44
11	495	94.34
12	660	94.09
13	858	93.70
14	1092	82.12
15	1365	79.96
16	1680	76.96
17	2040	72.84
18	2448	65.81
19	2907	68.04
20	3420	67.69
21	3990	68.25
22	4620	67.58
